# Oxygenation-Based Severity Stratification and a Proposed Clinical Diagnostic Workflow for Non-HIV *Pneumocystis jirovecii* Pneumonia: A Single-Center Observational Study

**DOI:** 10.3390/jof12070541

**Published:** 2026-07-22

**Authors:** Jing Chen, Min Wu, Zhonghua Deng, Yingqiu Ying, Ming Lu

**Affiliations:** 1 Department of Infectious Disease, Peking University Third Hospital, Beijing 100191, China; jchen@bjmu.edu.cn (J.C.); dengzhonghuatj@163.com (Z.D.); 2 Department of Pharmacy, Fuzhou Second General Hospital, Fuzhou 350007, China; wumin1539@163.com; 3 Department of Pharmacy, Peking University Third Hospital, Beijing 100191, China

**Keywords:** *Pneumocystis jirovecii* pneumonia, non-HIV, immunocompromised host, oxygenation, proposed clinical diagnostic workflow, bronchoalveolar lavage fluid, metagenomic next-generation sequencing, co-pathogen

## Abstract

Non-HIV *Pneumocystis jirovecii* pneumonia (PJP) is a life-threatening opportunistic fungal pneumonia that may progress rapidly in immunocompromised hosts. Broad bronchoalveolar lavage fluid (BALF) molecular testing supports microbiologic recognition, but additional organisms often require bedside adjudication. We conducted a single-center observational study of 49 HIV-negative adults with clinically confirmed PJP, routine BALF metagenomic next-generation sequencing support, and complete 30-day follow-up. Diagnosis required compatible symptoms and chest computed tomography findings, microbiologic support for *P. jirovecii*, and infectious disease specialist exclusion of isolated colonization. The primary endpoint was ICU-level care requirement, defined as ICU admission, invasive mechanical ventilation, or 30-day all-cause mortality. Recent immunosuppressive exposure was present in 48 patients (98.0%). ICU-level care was required in 15 patients (30.6%); all ventilation and death events occurred in this group, and 30-day mortality was 10.2%. Baseline PaO2/FiO2 < 200 mmHg was associated with higher proportions of ICU admission, mechanical ventilation, and death. Chronic kidney disease, lower creatinine clearance, higher lactate dehydrogenase, and bacterial co-pathogen context showed exploratory signals, whereas overall co-pathogen positivity was heterogeneous. These findings support integrating oxygenation status, host vulnerability, and conservative co-pathogen adjudication to guide escalation and antimicrobial decisions after BALF testing.

## 1. Introduction

*Pneumocystis jirovecii* pneumonia (PJP) remains a life-threatening opportunistic fungal pneumonia in immunocompromised patients. Although historically associated with advanced human immunodeficiency virus infection, PJP is increasingly recognized in HIV-negative patients receiving glucocorticoids, chemotherapy, calcineurin inhibitors, antimetabolites, biologics, or post-transplant immunosuppression [1,2,3,4].

Non-HIV PJP differs clinically from HIV-associated disease. Organism burden may be lower, inflammatory lung injury may be abrupt, and respiratory failure may progress rapidly. Contemporary definitions, treatment guidance, prevention guidance, and outcome studies emphasize the integration of host risk, compatible pulmonary disease, microbiologic evidence, oxygenation severity, and timely therapy rather than reliance on a single test result [5,6,7,8,9,10,11,12,13,14].

For clinicians managing immunocompromised pneumonia, broad respiratory molecular testing has created both opportunity and risk. Bronchoalveolar lavage fluid (BALF) metagenomic next-generation sequencing (mNGS) can provide microbiologic support for *P. jirovecii* and identify additional candidate organisms, but a reported organism does not automatically establish tissue-invasive disease, causal co-infection, or a need for additional antimicrobial therapy [15,16,17,18,19,20,21,22,23,24]. This distinction is particularly relevant when bacteria, cytomegalovirus (CMV), Aspergillus, or respiratory viruses are detected in profoundly immunocompromised hosts.

Most available molecular diagnostic studies emphasize diagnostic yield, test performance, or organism detection. A separate and clinically important question arises after *P. jirovecii* has been detected and the case is judged clinically compatible: how should clinicians triage severity, interpret additional BALF organisms, document prevention opportunities, and plan escalation when quantitative fungal burden data are unavailable? This gap is especially relevant for non-HIV PJP because deterioration may occur before confirmatory tests, quantitative burden measures, or full co-pathogen adjudication are complete.

Therefore, we framed this study as a clinical report-based observational study that proposes, but does not validate, a clinical diagnostic workflow with oxygenation-based severity stratification. We did not aim to evaluate mNGS diagnostic accuracy, organism-burden thresholds, or a multivariable prognostic model. Among HIV-negative immunocompromised patients with clinically confirmed PJP supported by routine BALF testing, we assessed how host phenotype, oxygenation-defined severity, treatment recognition timing, and clinician-adjudicated co-pathogen context characterized ICU-level care requirement.

## 2. Materials and Methods

### 2.1. Study Design and Population

We conducted a single-center observational study of HIV-negative patients with clinically confirmed PJP supported by routine BALF microbiologic testing at Peking University Third Hospital between January 2023 and December 2025. The analytic cohort comprised 49 patients with complete 30-day outcome information. Non-HIV status was defined by HIV-negative status in available clinical records. Immunocompromised status was defined by at least one of the following within 6 months before PJP diagnosis: solid organ transplantation, malignancy or hematologic disease, autoimmune disease or immunosuppressive-drug exposure, chronic glucocorticoid exposure, calcineurin inhibitor or antimetabolite exposure, biologic therapy, chronic kidney disease with immunosuppressive treatment, or another clinician-documented immunosuppressive condition. Reporting followed STROBE principles [25].

### 2.2. Clinical Diagnostic Workflow and Routine BALF mNGS Reporting

PJP diagnosis required compatible symptoms; compatible chest computed tomography (CT) findings, including bilateral ground-glass opacities or diffuse interstitial/alveolar infiltrates; microbiologic support for *P. jirovecii* from routine BALF mNGS; and infectious disease specialist exclusion of isolated colonization. BALF mNGS was a routine clinical test rather than a research sequencing assay performed by the investigators. Treating clinicians ordered the test when clinically indicated. BALF specimens were processed by the Department of Clinical Laboratory according to the laboratory clinical standard operating procedures, and formal microbiological reports were released to treating teams and retained in the medical record.

Specimens in the present cohort were submitted to the Department of Clinical Laboratory of Peking University Third Hospital for pathogenic mNGS using the PACEseq clinical reporting workflow (Hugobiotech Co., Ltd., Beijing, China). A related PJP NGS study from the same Department of Laboratory Medicine and Clinical Microbiology Sequencing Platform has described the hospital workflow in greater methodological detail [20]. In that workflow, nucleic acids from BALF or sputum submitted for targeted NGS were extracted from 200 µL samples using the Hugo Biotech YGZZ017 kit (Hugo Biotech Co., Ltd., Beijing, China); for metagenomic NGS, DNA was extracted using the QIAamp DNA Micro Kit and libraries were prepared using the QIAseq Ultralow Input Library Kit (QIAGEN, Hilden, Germany). Samples underwent single-end 75 bp sequencing on an Illumina NextSeq550Dx platform (Illumina, San Diego, CA, USA) [20].

Bioinformatic processing in the published hospital workflow included adapter and low-quality read removal using fastp version 0.20.0 for targeted NGS, host-read removal against the human genome GRCh38.101 using bowtie2, and microbial identification by BLAST against a local database derived from NCBI RefSeq and GenBank. For metagenomic NGS, sequences underwent quality filtering (Q ≥ 30 and length ≥ 35 bp), host sequence removal against GRCh38.101, and alignment to the NCBI Microbial Genome Database. Each batch included no-template controls consisting of sterile water and synthetic positive controls [20]. These details are provided to clarify the routine clinical laboratory reporting framework, whereas the present de-identified research dataset contained final clinical reports and patient-level clinical variables.

The formal reporting criteria used in the present study were consistent with the hospital workflow described above. For bacteria other than *Mycobacterium*, fungi other than *Cryptococcus*, and parasites, a positive result required either: (1) genome coverage of unique reads mapped to the target microorganism ranking among the top 10 organisms in the same microbial category, with the microorganism absent from the no-template control; or (2) RPM-r (RPMsample/RPMNTC) > 10 when RPMNTC was not zero. For *Mycobacterium tuberculosis* and *Cryptococcus spp.*, the published laboratory workflow used organism-specific criteria requiring at least one target-specific sequence absent from the no-template control or a sample-to-NTC RPM ratio > 5 when an NTC signal was present [20]. Reported candidate organisms were not interpreted as clinically causal by sequencing thresholds alone; potential contaminants, colonizers, and commensal organisms were further evaluated through the clinical adjudication process described below.

**Figure 1:**
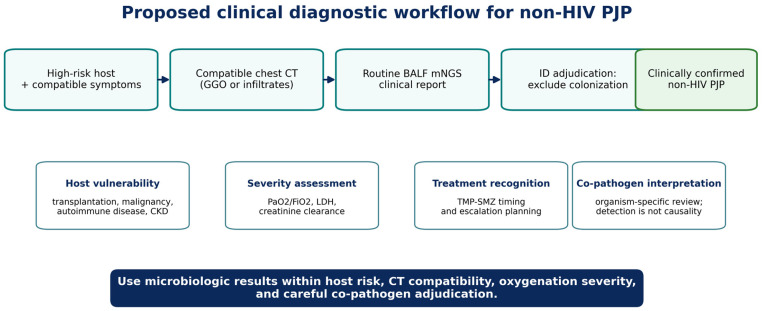
Proposed clinical diagnostic workflow and oxygenation-based severity stratification approach for clinically confirmed non-HIV PJP. The workflow integrates high-risk host status, compatible symptoms and CT findings, routine BALF microbiologic support, infectious disease adjudication to exclude isolated colonization, oxygenation-based severity assessment, treatment recognition timing, co-pathogen interpretation, and escalation/outcome planning. This workflow was derived from the present clinical cohort and has not been prospectively validated or compared with alternative diagnostic approaches. Abbreviations: BALF, bronchoalveolar lavage fluid; CKD, chronic kidney disease; CT, computed tomography; GGO, ground-glass opacity; ID, infectious diseases; LDH, lactate dehydrogenase; mNGS, metagenomic next-generation sequencing; PaO2/FiO2, ratio of arterial oxygen partial pressure to fractional inspired oxygen; PJP, *Pneumocystis jirovecii* pneumonia; TMP-SMZ, trimethoprim-sulfamethoxazole.

This study did not evaluate mNGS sensitivity, specificity, comparative diagnostic accuracy, sequencing depth, or normalized organism burden. Although the related laboratory workflow provides reproducible wet-laboratory and bioinformatics details for routine PJP NGS testing [20], patient-level quantitative read counts, reads per million values, organism-specific coverage depth, host-normalized microbial burden, total read depth, and raw FASTQ files from the present clinical cohort were not uniformly exported into the de-identified research dataset. Pneumocystis PCR was not performed, and serum beta-D-glucan was available only for a subset of patients. No post hoc organism burden cutoff was applied. Accordingly, the manuscript reports the clinical laboratory thresholds available in formal reports and the clinicians’ subsequent adjudication, rather than a new validation of BALF mNGS. The proposed clinical diagnostic workflow and severity stratification approach are summarized in Figure 1.

### 2.3. Co-Pathogen Adjudication

Candidate co-pathogens reported from BALF were not considered clinically causal by detection alone. Two infectious disease specialists, blinded to 30-day outcome status, reviewed symptoms, immune status, CT findings, inflammatory markers, conventional microbiology when available, antimicrobial decisions, treatment response, and temporal relationship to the pulmonary syndrome. Disagreements were resolved by consensus. Bacterial, CMV, acute SARS-CoV-2/COVID-19, influenza, and *Aspergillus* categories were retained as clinician-adjudicated context. CMV and Aspergillus were not equated with proven tissue-invasive CMV pneumonia or proven invasive aspergillosis without additional evidence [26,27,28,29].

### 2.4. Variables and Outcomes

Baseline severe PJP was defined as PaO2/FiO2 < 200 mmHg. Host phenotype variables included solid organ transplantation, specifically kidney transplantation in all cases, malignancy or hematologic disease, autoimmune or immunosuppressive drug category, non-transplant kidney disease, chronic kidney disease, diabetes mellitus, and renal rheumatic phenotype. After data review, the solid organ transplantation phenotype corresponded to kidney transplantation; the transplant category was, therefore, harmonized. Treatment variables included documented trimethoprim-sulfamethoxazole (TMP-SMZ) use and the interval from symptom onset to TMP-SMZ initiation. Adjunctive corticosteroid exposure for PJP was recorded as documented when either a corticosteroid agent or corticosteroid dose was present in the treatment fields. Corticosteroid use was determined by treating clinicians rather than assigned by a study protocol.

The primary clinical endpoint was ICU-level care requirement, defined a priori as ICU admission, invasive mechanical ventilation, or 30-day all-cause mortality. Thirty-day mortality was also reported separately. During analysis, this endpoint was operationally equivalent to ICU admission because all ventilation and death events occurred among ICU-admitted patients. It should, therefore, be interpreted as a pragmatic marker of critical care requirement rather than as a mechanistic prognostic composite.

### 2.5. Statistical Analysis

Categorical variables are reported as n/N (%) and continuous variables as median (interquartile range). Fisher exact tests and Mann–Whitney U tests were used for exploratory comparisons. Risk ratios were Haldane–Anscombe-corrected when sparse or zero cells were present. TMP-SMZ timing was analyzed using the cohort median interval from symptom onset to TMP-SMZ initiation (≤10 vs. >10 days). With only five deaths, multivariable regression was not performed to avoid sparse data bias, separation, and overfitting [30,31]. Correlation between normalized fungal burden and PaO2/FiO2 could not be evaluated because host-normalized mNGS burden metrics were unavailable. Effect estimates were interpreted as descriptive clinical signals, not independent predictors. All analyses were unadjusted and not corrected for multiplicity. Analyses were performed with R version 4.4.1.

## 3. Results

### 3.1. Cohort Profile

The cohort included 49 clinically confirmed non-HIV PJP patients with complete 30-day outcome information. Median age was 58.0 years, 28 patients (57.1%) were male, and 48 patients (98.0%) had recent immunosuppressive exposure. Glucocorticoid exposure within 6 months was recorded in 30 patients (61.2%). According to medication and clinical records, no patient received PJP prophylaxis before the PJP episode. TMP-SMZ therapy was documented in all 49 patients, and the median interval from symptom onset to TMP-SMZ initiation was 10.0 days. Adjunctive corticosteroid treatment during PJP management was documented in 47 patients (95.9%), including five of five non-survivors and 42 of 44 survivors (Table 1).

### 3.2. ICU-Level Care Requirement

**Figure 2:**
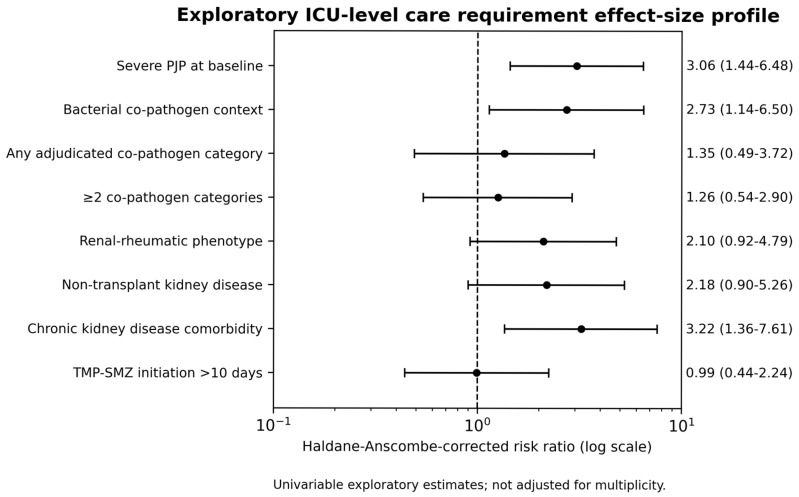
Exploratory ICU-level care requirement effect-size profile. Risk ratios were Haldane–Anscombe corrected. ICU-level care requirement was defined a priori as ICU admission, invasive mechanical ventilation, or 30-day mortality and was operationally equivalent to ICU admission in this cohort. Estimates are univariable and exploratory and should not be interpreted as independent predictors or as a multivariable ranking. Abbreviations: ICU, intensive care unit; PJP, *Pneumocystis jirovecii* pneumonia; TMP-SMZ, trimethoprim-sulfamethoxazole.

ICU-level care was required in 15 patients (30.6%). Because all invasive mechanical ventilation and death events occurred among ICU-admitted patients, this endpoint should be interpreted as critical care requirement rather than as an independent composite prognostic construct. ICU-level care requirement was more frequent among patients with oxygenation-defined severe PJP, chronic kidney disease, and bacterial co-pathogen context (Table 2). Continuous comparisons showed lower PaO2/FiO2, higher lactate dehydrogenase (LDH), and lower creatinine clearance among patients who required ICU-level care. The corresponding univariable effect-size profile is shown in Figure 2.

### 3.3. Oxygenation-Defined Severe PJP

Among 48 patients with recorded baseline severity, nine had severe PJP by PaO2/FiO2 < 200 mmHg. Compared with mild-to-moderate disease, severe PJP was associated with more frequent ICU admission, invasive mechanical ventilation, and 30-day death, as well as lower PaO2/FiO2 and lower creatinine clearance (Table 3). Severe PJP was not characterized by a higher overall burden of clinician-adjudicated co-pathogen categories. Adjunctive corticosteroid treatment was common across oxygenation strata: it was documented in 47/49 patients (95.9%) overall, including 9/9 patients (100.0%) with severe PJP, 37/39 patients (94.9%) with mild-to-moderate PJP, and the one patient with missing baseline severity classification. Therefore, corticosteroid exposure was not restricted to patients with low PaO2/FiO2, and no corticosteroid-treatment effect comparison was possible.

### 3.4. Treatment Timing and Co-Pathogen Context

Using the cohort median cutoff, TMP-SMZ initiation after 10 days did not show a worse ICU-level care gradient. This timing variable should be interpreted as a treatment-recognition pathway measure rather than a causal treatment effect variable because symptom onset, hospital admission, BALF sampling, laboratory reporting, and treatment initiation could not be decomposed into standardized intervals (Table S1).

At least one clinician-adjudicated co-pathogen category was present in 36 patients (73.5%). Bacterial co-pathogen context was the most frequent clinically actionable category (20/49, 40.8%), followed by CMV (15/49, 30.6%), acute SARS-CoV-2/COVID-19 (15/49, 30.6%), *Aspergillus* (3/49, 6.1%), and influenza virus (1/49, 2.0%) (Table S2). Host phenotype patterns were descriptive: renal rheumatic and chronic kidney disease phenotypes showed relatively high proportions of bacterial co-pathogen context and ICU-level care requirement, whereas deaths were uncommon in the harmonized kidney transplant group (Table S3). Detailed bacterial records are shown in Table S4.

## 4. Discussion

### 4.1. Main Findings and Contribution

This study proposes a clinical diagnostic workflow and oxygenation-based severity-stratification approach for clinically confirmed non-HIV PJP in immunocompromised hosts. Three observations are clinically relevant. First, impaired gas exchange was a clearer marker of ICU-level care requirement than the overall number of organisms reported from BALF testing. Second, kidney-related host vulnerability was linked to both severity and the complexity of antimicrobial management. Third, additional organisms detected by broad BALF testing required conservative adjudication rather than reflexive interpretation as causal co-infection.

Impaired oxygenation has been reported previously in severe non-HIV PJP and in immunocompromised acute hypoxemic respiratory failure [11,12,13,14]. The value of the present study lies in the clinical step after microbiologic recognition: when a routine BALF mNGS report supports *P. jirovecii* and also lists additional organisms, oxygenation status provides an immediate triage anchor while clinicians adjudicate which reported organisms require treatment, observation, or de-escalation. This framing is intended to make real-world clinical interpretation more transparent, not to replace quantitative microbiologic assessment where such data are available.

### 4.2. Oxygenation as a Triage Anchor in Non-HIV PJP

Oxygenation-defined severity was the clearest anchor for clinical interpretation. Patients with PaO2/FiO2 < 200 mmHg had higher proportions of ICU admission, invasive mechanical ventilation, and 30-day death; patients requiring ICU-level care also had lower PaO2/FiO2 and higher LDH. This is biologically plausible because non-HIV PJP can cause abrupt inflammatory lung injury despite lower organism burden, with alveolar-capillary barrier disruption, impaired surfactant function, and reduced oxygen reserve. In clinical practice, falling PaO2/FiO2 in an immunocompromised patient with compatible CT findings should prompt close monitoring, escalation planning, and reassessment for treatable co-pathogens or complications [11,12,13,14].

This interpretation is deliberately pragmatic. The endpoint was prespecified as ICU admission, invasive mechanical ventilation, or 30-day death, but its components overlapped completely with ICU admission in this cohort. We, therefore, interpret the endpoint as ICU-level care requirement rather than as a new prognostic construct. This distinction should reduce overstatement and makes the result more useful for clinicians: PaO2/FiO2 is not presented as an independent causal predictor, but as an accessible bedside measure that helps identify patients who need closer observation and earlier escalation planning.

### 4.3. Co-Pathogen Interpretation After Broad BALF Testing

The co-pathogen findings reinforce a central clinical interpretation principle: detection is not causality. Any clinician-adjudicated co-pathogen category was common, but the categories were heterogeneous. Overall co-pathogen positivity did not separate patients with and without ICU-level care requirement, whereas bacterial co-pathogen context showed an exploratory signal. This pattern argues against simple organism counting and supports organism-specific adjudication based on host risk, CT evolution, conventional microbiology, biomarkers, treatment response, and opportunities for antimicrobial de-escalation.

Bacterial co-pathogen context was more frequent among patients requiring ICU-level care and is clinically actionable, but this unadjusted signal may reflect true bacterial contribution, greater diagnostic intensity in sicker patients, treatment selection context, or lung injury that facilitates bacterial invasion. CMV, *Aspergillus*, SARS-CoV-2, and influenza require even more cautious interpretation. Detection should not trigger reflexive antiviral or antifungal treatment without supporting viral load, fungal biomarkers, imaging evolution, end-organ evidence, host-specific risk, or treatment response information [26,27,28,29,32,33,34,35,36,37].

### 4.4. Host Vulnerability, Prophylaxis Documentation, and Treatment Safety

Kidney-related host vulnerability was another clinically relevant signal. Chronic kidney disease and renal rheumatic phenotypes showed relatively high proportions of ICU-level care requirement and bacterial co-pathogen context. Renal dysfunction may alter antimicrobial pharmacokinetics, narrow the therapeutic window for TMP-SMZ, and amplify the consequences of fluid overload or systemic inflammation. Renal rheumatic diseases often require glucocorticoids, cytotoxic agents, or B-cell-depleting therapy, potentially impairing cellular immune control of *Pneumocystis*. Because these subgroups were small and overlapping, the findings are best interpreted as host vulnerability signals rather than independent risk factors.

LDH and creatinine clearance should be interpreted cautiously. Elevated LDH is compatible with pulmonary tissue injury in PJP but is not specific and may also reflect lung injury from co-pathogens, systemic inflammation, tissue injury, or other comorbid conditions. Reduced creatinine clearance may reflect pre-existing chronic kidney disease, acute kidney injury, or treatment-related physiology rather than PJP severity alone. These variables are, therefore, best treated as contextual bedside signals that may support, but should not replace, oxygenation assessment and clinical judgment.

### 4.5. Practical Bedside Implications

The findings can be translated into a proposed bedside workflow, provided the workflow is viewed as a structured interpretation aid rather than a validated rule. In an immunocompromised patient with compatible symptoms and CT findings, microbiologic support for *P. jirovecii* should be integrated with immediate oxygenation assessment rather than interpreted in isolation. PaO2/FiO2 should be documented at recognition and reassessed when respiratory support changes. Additional BALF organisms should be adjudicated one by one, with explicit reasons for treatment, observation, or de-escalation. Finally, prophylaxis eligibility and contraindications should be documented before discharge and when immunosuppression is continued or intensified.

This workflow has not been prospectively validated, has not been compared with alternative diagnostic approaches, and does not replace confirmatory diagnostics. Where available in clinical practice, Pneumocystis PCR, serum beta-D-glucan, quantitative mNGS metrics, fungal biomarkers, viral load testing, and conventional culture remain important. The present study instead addresses a common real-world situation in which some of these data are incomplete: clinicians must still decide how closely to monitor the patient, whether to escalate care, and how to avoid both undertreatment and unnecessary antimicrobial expansion.

### 4.6. Limitations

This study has limitations. It was single-center and retrospective, with 49 patients and five 30-day deaths. Small changes in event counts can, therefore, produce substantial percentage differences, and all subgroup comparisons should be interpreted cautiously. Multivariable modeling was not appropriate, and confounding by indication, illness severity, and diagnostic intensity could not be fully addressed.

BALF mNGS was performed through a routine-care clinical laboratory reporting workflow rather than as a study-specific research sequencing protocol. We expanded the Methods by incorporating the detailed workflow reported by a related PJP NGS study from the same Department of Laboratory Medicine and Clinical Microbiology Sequencing Platform [20]. However, the investigators of the present study did not perform BALF processing, nucleic acid extraction, library preparation, sequencing, or primary bioinformatic analysis. Final clinical reports were available, but patient-level quantitative read counts, raw sequence files, total read depth, RPM values, coverage depth, host DNA or RNA normalization, and organism burden were not uniformly available in the de-identified clinical dataset. Accordingly, normalized fungal burden and its correlation with PaO2/FiO2 could not be assessed.

Pneumocystis PCR was not performed, and serum beta-D-glucan was available only for a subset of patients, and the study was not designed to estimate diagnostic accuracy. Treatment variables beyond TMP-SMZ documentation, TMP-SMZ timing, and descriptive adjunctive corticosteroid documentation were incomplete. Adjunctive corticosteroid therapy was documented in almost all patients in the cohort, including all patients with PaO2/FiO2 < 200 mmHg and most patients with mild-to-moderate oxygenation status; however, dose, duration, and indication were not standardized, and the absence of a meaningful untreated comparator group precluded evaluation of corticosteroid treatment effect.

These limitations constrain causal inference and external generalizability. However, they do not negate the central clinical message: in clinically confirmed non-HIV PJP, broad microbiologic results are most useful when embedded in structured assessment of host vulnerability, CT compatibility, oxygenation severity, treatment recognition, and conservative infectious disease adjudication.

## 5. Conclusions

In clinically confirmed non-HIV PJP supported by routine BALF testing, oxygenation-defined severity was more clinically actionable than overall co-pathogen positivity for identifying patients requiring ICU-level care. We propose a clinical diagnostic workflow that integrates microbiologic support with host phenotype, CT compatibility, early and repeated oxygenation assessment, timely anti-PJP therapy, escalation planning, prophylaxis documentation, and conservative co-pathogen adjudication. This workflow should be interpreted as a hypothesis-generating clinical aid that requires prospective validation.

## Figures and Tables

**Table 1:** Cohort profile, treatment documentation, and key clinical variables.

Characteristic	Overall (N = 49)	30-Day Non-Survivors (n = 5)	30-Day Survivors (n = 44)
Age, years	58.0 (47.0–70.0)	65.0 (65.0–70.0)	57.0 (46.8–69.2)
Male sex	28/49 (57.1%)	3/5 (60.0%)	25/44 (56.8%)
BMI, kg/m^2^	23.2 (21.3–25.6)	25.4 (19.6–27.3)	23.1 (21.5–25.5)
Recent immunosuppressive exposure	48/49 (98.0%)	5/5 (100.0%)	43/44 (97.7%)
Glucocorticoids within 6 months	30/49 (61.2%)	3/5 (60.0%)	27/44 (61.4%)
Pre-episode PJP prophylaxis received	0/49 (0.0%)	0/5 (0.0%)	0/44 (0.0%)
TMP-SMZ treatment documented	49/49 (100.0%)	5/5 (100.0%)	44/44 (100.0%)
Symptom onset to TMP-SMZ, days	10.0 (7.0–19.0)	5.0 (5.0–10.0)	10.0 (7.0–19.5)
Alternative anti-PJP regimen documented in available treatment fields	0/49 (0.0%)	0/5 (0.0%)	0/44 (0.0%)
Adjunctive corticosteroid treatment documented	47/49 (95.9%)	5/5 (100.0%)	42/44 (95.5%)
Solid organ transplantation/kidney transplantation category	19/49 (38.8%)	1/5 (20.0%)	18/44 (40.9%)
Malignancy or hematologic disease category	18/49 (36.7%)	1/5 (20.0%)	17/44 (38.6%)
Renal rheumatic phenotype	5/49 (10.2%)	2/5 (40.0%)	3/44 (6.8%)
Chronic kidney disease comorbidity	18/49 (36.7%)	4/5 (80.0%)	14/44 (31.8%)
Bacterial co-pathogen category	20/49 (40.8%)	4/5 (80.0%)	16/44 (36.4%)
Any clinician-adjudicated co-pathogen category	36/49 (73.5%)	4/5 (80.0%)	32/44 (72.7%)
Severe PJP at baseline	9/48 (18.8%)	3/4 (75.0%)	6/44 (13.6%)
ICU admission	15/49 (30.6%)	5/5 (100.0%)	10/44 (22.7%)
Invasive mechanical ventilation	5/49 (10.2%)	3/5 (60.0%)	2/44 (4.5%)
PaO2/FiO2 at baseline	268.6 (215.2–363.8)	156.8 (136.9–195.2)	281.8 (226.6–366.7)
LDH, U/L	327.0 (240.0–464.0)	549.0 (464.8–587.2)	322.0 (239.0–403.0)
Creatinine clearance, mL/min	41.9 (23.9–57.4)	31.7 (23.6–40.0)	45.5 (24.5–59.5)

Values are n/N (%) or median (interquartile range). Percentages use available non-missing denominators unless otherwise specified. Adjunctive corticosteroid treatment was counted as documented when either a corticosteroid agent or corticosteroid dose was recorded in the treatment fields. PaO2/FiO2 summaries include numeric ratio values only. Pre-episode PJP prophylaxis was assessed from medication and clinical records and should be interpreted as a recorded treatment status variable rather than missing documentation. The transplant category was harmonized because solid-organ transplantation corresponded to kidney transplantation in this cohort. Abbreviations: BMI, body mass index; ICU, intensive care unit; LDH, lactate dehydrogenase; PaO2/FiO2, arterial oxygen partial pressure to fractional inspired oxygen ratio; PJP, *Pneumocystis jirovecii* pneumonia; TMP-SMZ, trimethoprim-sulfamethoxazole.

**Table 2:** Univariable exploratory comparisons for ICU-level care requirement.

Characteristic	ICU-Level Care If Variable Present	ICU-Level Care If Variable Absent	Corrected RR (95% CI)	Fisher or Mann-Whitney P
Severe PJP at baseline *	6/9 (66.7%)	8/39 (20.5%)	3.06 (1.44–6.48)	0.012
Bacterial co-pathogen category	10/20 (50.0%)	5/29 (17.2%)	2.73 (1.14–6.50)	0.026
Any adjudicated co-pathogen category	12/36 (33.3%)	3/13 (23.1%)	1.35 (0.49–3.72)	0.727
≥2 co-pathogen categories	5/14 (35.7%)	10/35 (28.6%)	1.26 (0.54–2.90)	0.735
Renal-rheumatic phenotype	3/5 (60.0%)	12/44 (27.3%)	2.10 (0.92–4.79)	0.160
Non-transplant kidney disease	2/3 (66.7%)	13/46 (28.3%)	2.18 (0.90–5.26)	0.218
Chronic kidney disease comorbidity	10/18 (55.6%)	5/31 (16.1%)	3.22 (1.36–7.61)	0.009
TMP-SMZ initiation >10 days	7/23 (30.4%)	8/26 (30.8%)	0.99 (0.44–2.24)	1.000
PaO2/FiO2 at baseline	209.8 (156.8–262.8) (n = 14)	289.0 (252.9–382.6) (n = 27)	NA	0.002
LDH, U/L	445.0 (301.0–601.0) (n = 13)	280.0 (229.0–375.5) (n = 32)	NA	0.006
CRP, mg/L	49.7 (25.0–87.1) (n = 13)	38.3 (23.8–60.1) (n = 33)	NA	0.479
Creatinine clearance, mL/min	23.5 (19.8–40.6) (n = 14)	47.4 (35.3–60.0) (n = 33)	NA	0.016
Symptom onset to TMP-SMZ, days	7.0 (5.5–45.0) (n = 15)	10.0 (7.0–18.5) (n = 34)	NA	0.711

ICU-level care requirement was defined as ICU admission, invasive mechanical ventilation, or 30-day death and was operationally equivalent to ICU admission. * The severe PJP comparison excludes one patient with missing baseline severity classification; therefore, the ICU-level care count in that row sums to 14 rather than 15. Corrected risk ratios and 95% confidence intervals were Haldane–Anscombe corrected. Comparisons are exploratory and not adjusted for multiplicity. Abbreviations: CI, confidence interval; CRP, C-reactive protein; ICU, intensive care unit; LDH, lactate dehydrogenase; NA, not applicable; PaO2/FiO2, ratio of arterial oxygen partial pressure to fractional inspired oxygen; PJP, *Pneumocystis jirovecii* pneumonia; RR, risk ratio; TMP-SMZ, trimethoprim-sulfamethoxazole.

**Table 3:** Clinical phenotype of oxygenation-defined severe PJP at baseline.

Characteristic	Severe PJP (n = 9)	Mild-to-Moderate PJP (n = 39)	Fisher or Mann-Whitney P
Bacterial co-pathogen category	5/9 (55.6%)	14/39 (35.9%)	0.451
Any adjudicated co-pathogen category	6/9 (66.7%)	29/39 (74.4%)	0.687
≥2 co-pathogen categories	1/9 (11.1%)	12/39 (30.8%)	0.410
Renal-rheumatic phenotype	1/9 (11.1%)	3/39 (7.7%)	1.000
Non-transplant kidney disease	0/9 (0.0%)	2/39 (5.1%)	1.000
ICU admission	6/9 (66.7%)	8/39 (20.5%)	0.012
Invasive mechanical ventilation	4/9 (44.4%)	1/39 (2.6%)	0.003
30-day death	3/9 (33.3%)	1/39 (2.6%)	0.017
Adjunctive corticosteroid treatment documented	9/9 (100.0%)	37/39 (94.9%)	1.000
PaO2/FiO2 at baseline	140.2 (114.0–157.6) (n = 9)	289.5 (252.9–375.9) (n = 32)	<0.001
LDH, U/L	412.5 (306.5–563.0) (n = 6)	311.5 (239.5–394.0) (n = 38)	0.177
CRP, mg/L	46.0 (26.4–94.8) (n = 8)	40.3 (23.8–66.0) (n = 37)	0.511
Creatinine clearance, mL/min	24.1 (16.9–35.4) (n = 7)	47.0 (33.6–60.7) (n = 39)	0.030
Symptom onset to TMP-SMZ, days	7.0 (5.0–15.0) (n = 9)	10.0 (7.0–20.0) (n = 39)	0.507

Severe PJP was defined as PaO2/FiO2 < 200 mmHg. One patient with missing baseline severity classification was excluded; severity-stratified outcome counts, therefore, sum to 48 patients, and one 30-day death occurred in the missing-severity patient. Adjunctive corticosteroid treatment was documented in 47/49 patients overall, including 9/9 patients with severe PJP, 37/39 patients with mild-to-moderate PJP, and the one patient with missing baseline severity classification. Because corticosteroid exposure was nearly universal and was not protocolized, no corticosteroid treatment effect comparison was possible. Continuous variables are median (interquartile range). Comparisons are exploratory and not adjusted for multiplicity. Abbreviations: CRP, C-reactive protein; ICU, intensive care unit; LDH, lactate dehydrogenase; PaO2/FiO2, arterial oxygen partial pressure to fractional inspired oxygen ratio; PJP, Pneumocystis jirovecii pneumonia; TMP-SMZ, trimethoprim-sulfamethoxazole.

## Data Availability

The de-identified clinical dataset supporting the findings of this study is available from the corresponding author upon reasonable request and subject to institutional and ethics requirements. Data are not publicly available because of patient privacy and institutional data governance restrictions. A related laboratory-based PJP NGS study from the same Department of Laboratory Medicine deposited raw sequence data in the Genome Sequence Archive under accession CRA020282. The present study, however, was an independent retrospective clinical report-based analysis and did not generate a separate research sequencing dataset for deposition. Raw sequence files and sample-level sequencing metadata from the present clinical cohort were not exported into the de-identified research dataset and may contain human genetic material and other privacy-sensitive information.

## Supplementary Materials

The following supporting information can be downloaded at: https://www.mdpi.com/article/10.3390/jof12070541/s1, Table S1: TMP-SMZ treatment-recognition timing analysis using the cohort median cutoff; Table S2: clinician-adjudicated co-pathogen categories retained as clinical diagnostic context; Table S3: host phenotype, co-pathogen context, and clinical course matrix; Table S4: detailed bacterial pathogen records retained for clinically adjudicated bacterial co-pathogen context.
