# Supplementary material for: Oxygenation-Based Severity Stratification and a Proposed Clinical Diagnostic Workflow for Non-HIV Pneumocystis jirovecii Pneumonia: A Single-Center Observational Study
Source: J Fungi (Basel). 2026 Jul 22;12(7):541. doi: 10.3390/jof12070541 (PMC13413413; doi:10.3390/jof12070541)
Supplement: Supplementary file 1 [file jof-12-00541-s001.zip › jof-4414752-supplementary.pdf]

Supplementary Material

Oxygenation-Based Severity Stratification and a Proposed Clinical Diagnostic Workflow for Non-HIV *Pneumocystis jirovecii* Pneumonia: A Single-Center Observational Study

Supplementary Tables S1-S4

Table S1. TMP-SMZ treatment-recognition timing analysis using the cohort median cutoff.

Characteristic	TMP-SMZ ≤10 days (n=26)	TMP-SMZ >10 days (n=23)	Fisher or Mann-Whitney P
Severe PJP at baseline	6/26 (23.1%)	3/23 (13.0%)	0.472
ICU admission	8/26 (30.8%)	7/23 (30.4%)	1.000
Invasive mechanical ventilation	3/26 (11.5%)	2/23 (8.7%)	1.000
30-day death	3/26 (11.5%)	2/23 (8.7%)	1.000
ICU-level care requirement	8/26 (30.8%)	7/23 (30.4%)	1.000
Adjunctive corticosteroid treatment documented	25/26 (96.2%)	22/23 (95.7%)	1.000
Bacterial co-pathogen category	10/26 (38.5%)	10/23 (43.5%)	0.777
≥2 co-pathogen categories	4/26 (15.4%)	10/23 (43.5%)	0.055
PaO_2/FiO_2 at baseline	263.6 (188.6-336.8) (n=23)	287.6 (220.9-362.9) (n=18)	0.684
LDH, U/L	342.0 (239.5-474.0) (n=23)	311.5 (253.0-385.0) (n=22)	0.699
CRP, mg/L	53.4 (27.6-82.4) (n=24)	31.6 (22.9-47.7) (n=22)	0.073
Creatinine clearance, mL/min	45.5 (28.8-51.6) (n=25)	39.0 (23.0-63.3) (n=22)	0.907

TMP-SMZ timing was defined as the interval from symptom onset to TMP-SMZ initiation and should be interpreted as a treatment-recognition pathway measure rather than a causal treatment-effect variable. Adjunctive corticosteroid treatment was documented in 47/49 patients (95.9%) overall, including 9/9 patients (100.0%) with oxygenation-defined severe PJP, 37/39 patients (94.9%) with mild-to-moderate PJP, and the one patient with missing baseline severity classification. Because corticosteroid treatment was nearly universal, not assigned by a study protocol, and not standardized for dose or duration, it should not be interpreted as a comparative treatment exposure. Comparisons are exploratory and not adjusted for multiplicity. Abbreviations: CRP, C-reactive protein; ICU, intensive care unit; LDH, lactate dehydrogenase; PaO_2/FiO_2, ratio of arterial oxygen partial pressure to fractional inspired oxygen; PJP, *Pneumocystis jirovecii* pneumonia; TMP-SMZ, trimethoprim-sulfamethoxazole.

Table S2. Clinician-adjudicated co-pathogen categories retained as clinical diagnostic context.

Characteristic	Prevalence	30-day mortality within category
Any clinician-adjudicated co-pathogen category	36/49 (73.5%)	4/36 (11.1%)
Bacterial co-pathogen category	20/49 (40.8%)	4/20 (20.0%)
CMV co-pathogen category	15/49 (30.6%)	1/15 (6.7%)
Acute SARS-CoV-2/COVID-19	15/49 (30.6%)	0/15 (0.0%)
Aspergillus co-pathogen category	3/49 (6.1%)	0/3 (0.0%)
Influenza virus detected by BALF mNGS	1/49 (2.0%)	0/1 (0.0%)
0 co-pathogen categories	13/49 (26.5%)	1/13 (7.7%)
1 co-pathogen category	22/49 (44.9%)	3/22 (13.6%)
2 co-pathogen categories	11/49 (22.4%)	1/11 (9.1%)
≥3 co-pathogen categories	3/49 (6.1%)	0/3 (0.0%)

Categories are not mutually exclusive. Clinical adjudication does not automatically prove tissue-invasive disease. These categories were retained as context for clinical interpretation rather than as primary prognostic exposures. Abbreviations: BALF, bronchoalveolar lavage fluid; CMV, cytomegalovirus; mNGS, metagenomic next-generation sequencing; SARS-CoV-2, severe acute respiratory syndrome coronavirus 2.

Table S3. Host phenotype, co-pathogen context, and clinical course matrix.

Host phenotype	N	Bacteria	CMV	SARS-CoV-2/COVID-19	≥2 categories	ICU-level care requirement	30-day death
Solid organ transplantation/kidney transplantation	19	6/19 (31.6%)	7/19 (36.8%)	9/19 (47.4%)	6/19 (31.6%)	6/19 (31.6%)	1/19 (5.3%)
Malignancy/hematologic disease	18	7/18 (38.9%)	6/18 (33.3%)	5/18 (27.8%)	6/18 (33.3%)	2/18 (11.1%)	1/18 (5.6%)
Lymphoma/leukemia/multiple myeloma	11	5/11 (45.5%)	3/11 (27.3%)	3/11 (27.3%)	3/11 (27.3%)	1/11 (9.1%)	0/11 (0.0%)
Autoimmune/immunosuppressive-drug category	8	4/8 (50.0%)	3/8 (37.5%)	1/8 (12.5%)	3/8 (37.5%)	3/8 (37.5%)	1/8 (12.5%)
Non-transplant kidney disease	3	2/3 (66.7%)	1/3 (33.3%)	0/3 (0.0%)	1/3 (33.3%)	2/3 (66.7%)	2/3 (66.7%)
Renal-rheumatic phenotype	5	4/5 (80.0%)	1/5 (20.0%)	0/5 (0.0%)	1/5 (20.0%)	3/5 (60.0%)	2/5 (40.0%)
Chronic kidney disease comorbidity	18	11/18 (61.1%)	6/18 (33.3%)	5/18 (27.8%)	6/18 (33.3%)	10/18 (55.6%)	4/18 (22.2%)

Host phenotypes are not mutually exclusive. Co-pathogen categories are BALF-reported and clinician-adjudicated. The transplant phenotype was harmonized as solid organ transplantation/kidney transplantation after classification review. ICU-level care requirement was operationally equivalent to ICU admission. Adjunctive corticosteroid treatment was nearly universal in the cohort and is therefore not presented as a host-phenotype exposure in this matrix. Abbreviations: BALF, bronchoalveolar lavage fluid; CKD, chronic kidney disease; CMV, cytomegalovirus; ICU, intensive care unit; SARS-CoV-2, severe acute respiratory syndrome coronavirus 2.

Table S4. Detailed bacterial pathogen records retained for clinically adjudicated bacterial co-pathogen context.

Clinically adjudicated bacterial pathogen/record from BALF report	Number of records	Record-linked 30-day deaths
Bacterial co-pathogen category without species-level annotation	12	3
Pseudomonas aeruginosa	3	0
Methicillin-resistant *Staphylococcus aureus* (MRSA)	1	1
Streptococcus pneumoniae	1	1
Klebsiella pneumoniae	1	0
Staphylococcus aureus	2	0
Acinetobacter baumannii	1	0
Stenotrophomonas maltophilia	1	0
Escherichia coli	1	0
Mycoplasma	1	0

Rows are pathogen records, not mutually exclusive patient counts; some patients had more than one bacterial organism recorded. Record-linked deaths should not be summed across rows and may exceed patient-level deaths in the bacterial co-pathogen category. Cases without species-level annotation were retained only when the report and clinical context supported pathogenic or plausibly contributory bacterial co-pathogen status. Scientific names of bacterial and fungal organisms are italicized where applicable. Abbreviations: BALF, bronchoalveolar lavage fluid; MRSA, methicillin-resistant *Staphylococcus aureus*.
